# Pediatric dilated cardiomyopathy caused by TNNT2‐R151W mutation: Modeling and rescue in patient‐derived induced pluripotent stem cells and engineered heart tissue

**DOI:** 10.1002/btm2.70108

**Published:** 2026-01-06

**Authors:** Toshiaki Nagashima, Kenji Miki, Masaru Tsuchida, Julia Whitehouse, Ikue Takei‐Sasozaki, Yuki Higashiyama, Hidekazu Ishida, Kunio Kashino, Shigeru Miyagawa

**Affiliations:** ^1^ Department of Cardiovascular Surgery The University of Osaka Graduate School of Medicine Osaka Japan; ^2^ Premium Research Institute for Human Metaverse Medicine, The University of Osaka Osaka Japan; ^3^ Institute for Protein Research, The University of Osaka Osaka Japan; ^4^ Media Information Research Department NTT Communication Science Laboratories Kanagawa Japan; ^5^ Department of Pediatrics The University of Osaka Graduate School of Medicine Osaka Japan

**Keywords:** dilated cardiomyopathy, disease model, engineered heart tissue, iPSCs, pediatrics, TNNT2‐R151W mutation

## Abstract

Dilated cardiomyopathy (DCM) is the most prevalent pediatric cardiomyopathy and has a poor prognosis. Although heart transplantation is the only curative option, the severe donor shortage in Japan underscores an urgent need for alternative therapies. Here, we generated induced pluripotent stem cell (iPSC) lines from two unrelated children with the same TNNT2‐R151W mutation. We then investigated the cellular characteristics of the cardiomyocytes derived from these iPSC lines (R151W iPSC‐CMs) and evaluated their contractile function in pillar‐based engineered heart tissue (EHT). We also assessed the therapeutic potential of overexpressing wild‐type TNNT2 in patient‐derived iPSCs (wtTNNT2‐OE‐iPSCs). R151W‐iPSC‐CMs exhibited pronounced sarcomere disarray, attenuated Ca^2+^ transient amplitude, prolonged time to peak, and delayed decay tau. These characteristics are indicative of failing myocardium and were restored in wtTNNT2‐OE‐iPSC‐CMs. Pillar‐based EHT assays revealed a substantial decrease in contractile force in R151W‐EHTs compared to wtTNNT2‐OE‐EHTs, which is suggestive of the systolic dysfunction observed clinically in DCM. Collectively, these results provide the first functional evidence that the TNNT2‐R151W mutation leads to pediatric DCM by causing sarcomere insufficiency and disturbances in Ca^2+^ handling. Our patient‐specific iPSC‐EHT platform faithfully recapitulates key features of pediatric DCM and could offer a robust system for mechanistic studies and drug testing. Furthermore, phenotypic rescue upon overexpression of wild‐type TNNT2 suggests that this allele is amenable to a gene replacement approach aimed at restoring wild‐type function in TNNT2‐mutant cardiomyopathies.

**Translational Impact Statement**

This study generates a patient‐specific induced pluripotent stem cell (iPSC) and engineered heart tissue model of pediatric dilated cardiomyopathy caused by the TNNT2‐R151W mutation. This platform recapitulates key disease features, including sarcomere disarray, Ca^2+^ desensitization, and reduced contractile force, and demonstrates phenotypic rescue through the overexpression of wild‐type TNNT2. These findings functionally validate a high‐risk TNNT2 variant, support TNNT2‐directed gene replacement therapy, and provide a scalable, human system for the preclinical testing of precision therapies for pediatric cardiomyopathy.

## 1 INTRODUCTION

Dilated cardiomyopathy (DCM) is characterized by left ventricular dilatation and systolic dysfunction despite the absence of abnormal loading conditions such as hypertension, valve disease, or ischemic heart disease.^1^ It is the most common form of cardiomyopathy in children.^2^ According to epidemiological studies from the National Australian Childhood Cardiomyopathy Study +and the North American Pediatric Cardiomyopathy Registry, the incidence of newly diagnosed pediatric DCM varies by age group, ranging from 0.57 cases per 100,000 population per year among children aged 0 to 18 years,^3^ to 0.73 cases per 100,000 population per year in those ages 0 to 10 years.^2^ The survival free from death or transplantation is 74% 1 year after diagnosis, 62% at 10 years, and 56% at 20 years.^4^ Although pediatric DCM is associated with a poor prognosis, heart transplantation remains the only definitive treatment currently available. However, alternative therapies are urgently needed in Japan, where donors are severely limited.

Recent advances in genetic analysis have facilitated the identification of numerous causative genes.^5^, ^6^, ^7^, ^8^ These include genes encoding sarcomeric proteins such as cardiac troponin T (TNNT2),^9^ cardiac troponin I (TNNI3),^7^ and titin (TTN)^10^ as well as genes encoding non‐sarcomeric proteins such as lamin A/C (LMNA), which is involved in nuclear stability.^11^ Among the identified genetic variants, mutations in TNNT2 are the most prevalent and associated with more severe clinical outcomes.^12^ However, significant progress has yet to be achieved in elucidating the pathophysiology of DCM or in developing novel therapeutic strategies.

With the advent of induced pluripotent stem cells (iPSCs) technologies,^13^, ^14^ functional cardiomyocytes (CMs) can be obtained in vitro through the differentiation of human iPSCs.^15^, ^16^ To date, in vitro models using iPSC‐CMs derived from patients with various types of cardiac defects, such as long QT syndrome, Leopard syndrome, and Timothy syndrome have been reported.^17^, ^18^, ^19^ There are also numerous reports on DCM patient‐derived iPSCs,^20^, ^21^, ^22^, ^23^ with Sun et al. reporting generation of iPSC‐CMs with a missense mutation (R173W) in the TNNT2 gene from a family with DCM. When compared to iPSC‐CMs generated from healthy individuals in the same family cohort,^20^ the results showed that R173W iPSC‐CMs exhibited reduced calcium (Ca^2+^) transients, contractility, and abnormal sarcomeric α‐actinin distribution, which are characteristic of DCM phenotypes. Thus, iPSC‐CMs from DCM patients reproduce the DCM phenotypes morphologically and functionally and can be useful for exploring molecular and cellular mechanisms and optimizing the treatment of DCM.

Engineered heart tissue (EHT) using iPSC‐CMs is a promising platform that mimics the three‐dimensional structure and mechanical properties of native myocardium.^24^ EHT promotes the maturation of iPSC‐CMs more effectively than traditional two‐dimensional cultures, enabling the measurement of force generation under controlled mechanical loads.^25^ This allows for a more detailed evaluation of contraction dynamics associated with genetic cardiomyopathies, such as DCM.^23^, ^26^, ^27^ Furthermore, EHT systems offer an advanced in vitro platform for preclinical drug screening and mechanistic studies, bridging the gap between cellular phenotypes and organ‐level cardiac dysfunction.^28^ Therefore, EHT fabricated from patient‐specific iPSC‐CMs could be utilized as a disease model for evaluating the mechanical properties of pediatric DCM.

Here, we generated iPSC lines from two unrelated pediatric patients carrying the identical TNNT2‐R151W mutation and investigated the characteristics of patient‐specific iPSC‐CMs in vitro by comparing them with iPSC lines overexpressing wild‐type TNNT2 derived from patient‐specific iPSC lines. Furthermore, we fabricated EHT using iPSC‐CMs and evaluated their contractile dynamics in vitro.

## 2 MATERIALS AND METHODS

### 2.1 Human samples

We collected clinical data and biological samples from two DCM patients with the approval of the Ethics Committee of The University of Osaka Hospital (Ethics Review Approval Number 15211). Prior to blood sampling, we obtained written informed consent from a parent or legal guardian of the patients for both sample collection and genomic analysis. This study was conducted in accordance with the Ethical Guidelines for Medical and Health Research Involving Human Subjects in Japan and the principles outlined in the Declaration of Helsinki.

### 2.2 Generation and characterization of iPSCs and TNNT2 overexpressing iPSCs from two pediatric DCM patients

iPSCs were generated from peripheral blood mononuclear cells (PBMCs) obtained from two unrelated pediatric patients diagnosed with DCM, both heterozygous for the same TNNT2 mutation (c.451C>T, p.R151W). In brief, PBMCs were isolated from whole blood using Histopaque‐1083 (Sigma‐Aldrich) and reprogrammed into iPSCs using Sendai virus vectors (CytoTune‐iPS 2.0 Sendai Reprogramming Kit; Thermo Fisher Scientific) according to the manufacturer's instructions. Generated iPSCs were maintained on an iMatrix‐511 (Nippi)‐coated plate in StemFit AK02N medium (Ajinomoto) as previously described.^29^ The characterization of the generated iPSCs was confirmed by assessing pluripotency markers (OCT3/4, SOX2, and SSEA4) using flow cytometry (BD Biosciences), evaluating chromosomal abnormality by G‐banded karyotyping (Tottori Bioscience Promotion Foundation), and verifying the presence of the heterozygous TNNT2 mutation (c.451C>T) by Sanger sequencing (CoMIT Omics Center, The University of Osaka).

To generate iPSC lines stably expressing normal TNNT2, vectors were constructed using the PiggyBac transposon system (VectorBuilder). iPSCs were dissociated as described above and seeded onto iMatrix‐coated six‐well plates at a density of 1 × 10^5^ cells/well in StemFit AK02N medium supplemented with 10 μM Y‐27632 (ROCK inhibitor). The following day, cells were co‐transfected with the TNNT2 expression vector and the Super PiggyBac Transposase Expression Vector (System Biosciences, SBI) using FuGENE® HD Transfection Reagent (Promega) according to the manufacturer's instructions. Two days after the transfection, the EGFP‐positive cells were sorted and seeded onto laminin‐coated 6 cm dishes at a density of 300 or 600 cells per dish. After 8–10 days of culture, individual colonies were manually picked, and stable iPSC lines harboring the integrated target gene were established.

### 2.3 Cardiac differentiation

iPSCs were dissociated into single cells using a 1:1 dilution of 1 × TrypLE Select (Thermo Fisher Scientific) and 0.5 mM EDTA. The cells were then suspended at a density of 2 × 10^6^ cells per well in 1.5 mL of StemPro‐34 medium (Thermo Fisher Scientific) supplemented with 2 mM L‐glutamine (Thermo Fisher Scientific), 50 μg/mL ascorbic acid (Sigma), 0.4 mM monothioglycerol (MTG; Sigma), 150 μg/mL transferrin (Wako), 10 μM Y‐27632 (ROCK inhibitor; Fujifilm), 0.5% Matrigel (Corning), and 2 ng/mL BMP4 (R&D Systems). The cells were seeded in six‐well ultra‐low attachment plates (Corning) to initiate embryoid body (EB) formation. On day 1, an additional 1.5 mL of StemPro‐34 medium containing 2 mM L‐glutamine, 50 μg/mL ascorbic acid, 0.4 mM MTG, 150 μg/mL transferrin, 10 ng/mL basic fibroblast growth factor (bFGF; final concentration of 5 ng/mL), 12 ng/mL activin A (final concentration of 6 ng/mL), and 18 ng/mL BMP4 (final concentration of 10 ng/mL) were added. On day 3, the EBs were washed once with Iscove's Modified Dulbecco's Medium (IMDM; Thermo Fisher Scientific) and cultured in 3 mL of fresh StemPro‐34 medium containing 2 mM L‐glutamine, 50 μg/mL ascorbic acid, 0.4 mM MTG, 150 μg/mL transferrin, 10 ng/mL vascular endothelial growth factor (VEGF; R&D Systems), 1 μM IWP‐3 (Stemgent), 5.4 μM SB431542 (Sigma), and 0.6 μM dorsomorphin (Sigma). On day 6, the medium was changed to 3 mL of StemPro‐34 medium supplemented with 2 mM L‐glutamine, 50 μg/mL ascorbic acid, 0.4 mM MTG, 150 μg/mL transferrin, and 5 ng/mL VEGF. After that, the medium was changed to the same medium every 2–3 days. The cells were maintained under hypoxic conditions (5% O₂) for the first 10 days and then transferred to normoxic conditions for the remainder of the differentiation period.

### 2.4 Simple western

Proteins were extracted using RIPA buffer (Santa Cruz Biotechnology) according to the manufacturer's recommendation, and protein concentrations were measured using a NanoDrop 2000 spectrophotometer (Thermo Fisher Scientific). To analyze lysates on the JESS Simple Western™ instrument (ProteinSimple®), samples were diluted to total protein concentrations of 5 ng/μL in 1× fluorescent master mix (EZ standard pack I; ProteinSimple®), and 3 μL was added per well. Primary antibodies were used: anti‐GAPDH (Santa Cruz Biotechnology, sc‐47,724, 1:250), anti‐TNNT2 (Abcam, ab45932, 1:50), anti‐TNNI1 (Abcam, ab203515, 1:100), and anti‐TNNI3 (Proteintech, 21,652‐1‐AP, 1:250). Secondary antibodies were used: anti‐mouse antibody (ProteinSimple®, 042–205) and anti‐rabbit antibody (ProteinSimple®, 042–206). The Ab diluent, washing buffer, plates, and capillary cartridges used were derived from the 12–230 kDa separation module (ProteinSimple®).

### 2.5 Flow cytometric analysis

iPSCs were fixed and permeabilized using the BD Cytofix/Cytoperm™ Fixation/Permeabilization Kit (BD Biosciences). To identify pluripotent iPSCs, iPSCs were stained with OCT3/4 (BD Biosciences, 1:10), SOX2 (BD Biosciences, 1:30), and SSEA4 (BD Biosciences, 1:10).

Differentiated EBs were treated with 2 mg/mL collagenase type I for 1 h and then treated with 0.5 × TrypLE Select at 37°C for 15–20 min. To neutralize trypsin, 10% fetal bovine serum (FBS)/IMDM was added to the EBs to dissociate them into single cells, and then the cells were centrifuged at 1200 rpm for 5 min. We analyzed the CMs using FACS Canto II (BD Biosciences) and sorted the CMs using FACSAria Fusion (BD Biosciences). FACSDiva software (BD Biosciences) and FlowJo v10.10 software (BD Life Sciences) were used for analysis. For ACTN2 staining, cells were fixed and permeabilized in the same manner as the aforementioned and subsequently stained with purified ACTN2 (Abcam, ab68167; 1:200). As the secondary antibody, Goat anti‐mouse Alexa Fluor 647 was used (Thermo Fisher Scientific, A21235; 1:300). For calcium transients and immunocytochemistry experiments, cells were sorted based on CM and non‐CM surface markers. CMs were identified using PE/Cy7‐conjugated anti‐SIRPα (BioLegend, 323,808; 1:200), while non‐CM populations were labeled with APC‐conjugated anti‐CD90 (BD Biosciences, 559,869; 1:500), APC‐conjugated anti‐human CD31 (BioLegend, 303,116; 1:200), Alexa Fluor 647‐conjugated anti‐CD49a (BioLegend, 328,310; 1:200), and APC‐conjugated anti‐CD140b (BioLegend, 323,608; 1:200), as previously described.^30^, ^31^

### 2.6 Immunocytochemistry

The sorted CMs were seeded onto a fibronectin‐coated 35 mm dish (Thermo Fisher Scientific) and cultured for 3–4 days. The CMs were fixed with 4% paraformaldehyde (PFA) for 20 min, permeabilized in PBS containing 0.1% Triton X‐100 for 15 min and subsequently blocked with PBS supplemented with 0.1% Triton X‐100 and 2% donkey serum at room temperature for 1 h. The CMs were incubated overnight at 4°C with primary antibodies against cardiac Troponin T (cTNT) (BD Pharmingen, 564,766; 1:200) and ACTN2 (Abcam, ab68167; 1:200). The following day, the CMs were washed twice with PBS and incubated with Alexa Fluor 488‐conjugated donkey anti‐mouse IgG (Thermo Fisher Scientific; 1:500) and Alexa Fluor 555‐conjugated donkey anti‐rabbit IgG (Thermo Fisher Scientific; 1:500) for 1 h at room temperature in the dark. Nuclei were counterstained with Hoechst 33342 (DOJINDO) for 5 min under light‐protected conditions. Fluorescence images were acquired using a confocal laser scanning microscope (AX‐R, Nikon) and an inverted microscope (Ti2‐E, Nikon), both equipped with an Apochromat Lambda S 60 × oil‐immersion objective lens (NA 1.40, Nikon) and a motorized precision scanning stage. Image analysis was performed using NIS‐Elements software (Nikon). Sarcomere abnormality is defined by the presence of ACTN2 aggregation. For quantification, 50 cells per sample were analyzed from three independent experiments for each iPSC‐CM line (for a total of 150 cells per line). Images were acquired from randomly selected fields to avoid selection bias. The proportion of cells with ACTN2 aggregates was then calculated for each line. EHTs were fixed in 4% PFA for 2–3 h, rinsed with PBS, and immersed in 15% sucrose in PBS overnight at 4°C. The following day, samples were transferred to 30% sucrose in PBS and incubated for an additional 24 h at 4°C. EHTs were embedded in Tissue‐Tek Optimal Cutting Temperature (OCT) compound (Sakura Finetek Japan) using cryomolds (Sakura Finetek Japan) and cryosectioned into 5‐μm slices using a cryostat (Leica Biosystems). Subsequently, immunostaining images for ACTN2 and cTNT were obtained within the EHTs using the same protocol described above for CMs.

### 2.7 Ca^2+^ handling

The sorted CMs were plated at a density of 5 × 10^4^ cells in 5 μL of medium at the center of fibronectin‐coated 35 mm glass‐bottom dishes (Iwaki). After 1 h of cell attachment, additional medium was added. The culture medium was changed every 2 days. The CMs were incubated with Calbryte™ 590 AM (AAT Bioquest) for calcium imaging according to the manufacturer's instructions. Live‐cell fluorescence imaging was performed using a confocal laser scanning microscope (AX‐R, Nikon) and an inverted microscope (Ti2‐E, Nikon) equipped with an Apochromat Lambda S 25× oil‐immersion objective lens and a motorized precision scanning stage. During imaging, cells were maintained at 37°C under normoxic conditions (95% air, 5% CO₂) using a stage‐top incubator (STXG‐WSKMX‐SET, Tokai Hit). Fluorescence signals were acquired and initially processed using NIS‐Elements software (Nikon), followed by further quantitative analysis using MATLAB (MathWorks).

### 2.8 Fabrication of EHT and force analysis

The EHTs were fabricated as previously described.^32^ Briefly, differentiated EBs were dissociated in the same manner as for flow cytometric analysis. Then, the cells were resuspended at a density of 2 × 10^7^ cells/mL in a hydrogel solution prepared by combining StemPro‐34 medium supplemented with 2 mM L‐glutamine, 50 μg/mL ascorbic acid, 0.4 mM MTG, 150 μg/mL transferrin, and 5 ng/mL VEGF, along with fibrinogen (Sigma, final concentration of 5 mg/mL), Matrigel (Corning, final concentration of 5%), and aprotinin (Sigma, final concentration of 5 μg/mL). Thrombin (Sigma, final concentration of 2.5 U/mL) was then added to the cell–hydrogel mixture (total volume of 200 μL), which was immediately cast into the PDMS pillar molds and incubated at 37°C for 1 h to allow gelation. The fabricated EHTs were transferred into 24‐well culture plates and maintained in medium supplemented with 5 μg/mL aprotinin. The culture medium was replaced every 2 days. To assess the contractile force of the EHTs, the contractile dynamics of the EHTs under 1 Hz stimulation using a microscope system (BZ‐9000, Keyence) were recorded. Pillar deflection was measured using displacement data extracted via custom Python scripts. The contractile force was subsequently calculated using MATLAB software (MathWorks). The force generated by EHTs was estimated by modeling each pillar as a cantilever beam subjected to a concentrated load at the free end. The resulting deflection (δ) was calculated using the following equation:

$$\delta =\frac{PL^{3}}{3\mathrm{EI}},$$

$$I=\frac{\pi }{64}d^{4},$$

where *P* denotes the applied force, *L* is the length of the pillar (12 mm), *E* is the Young's modulus of PDMS (1.7 MPa), *d* is the pillar diameter (1.2 mm), and *I* represents the area moment of inertia.

Tissue displacement, reflecting the degree of pillar deflection, was quantified from digital video recordings acquired at a spatial resolution of 3.79 μm/pixel. Frame‐to‐frame displacement was analyzed using the phase‐only correlation (POC) method (Takita et al., 2003), a template‐matching image registration technique capable of detecting subpixel displacements using phase information alone. This approach is highly robust to variations in image brightness. Given that inter‐frame deformation during EHT contraction was minimal, POC enabled accurate and reliable motion tracking throughout the recording.

### 2.9 Statistical analysis

All data are presented as mean ± standard error of the mean (SEM) with three or more biological replicates, as stated in individual figure legends. Statistical analyses were performed using GraphPad Prism 10.4.0 (GraphPad, Inc., California, USA). Comparisons between two groups were conducted using unpaired two‐tailed Student's *t* tests where appropriate.

## 3 RESULTS

### 3.1 Generation of disease‐specific iPSCs and TNNT2 overexpression iPSCs from two pediatric DCM patients

**FIGURE 1:**
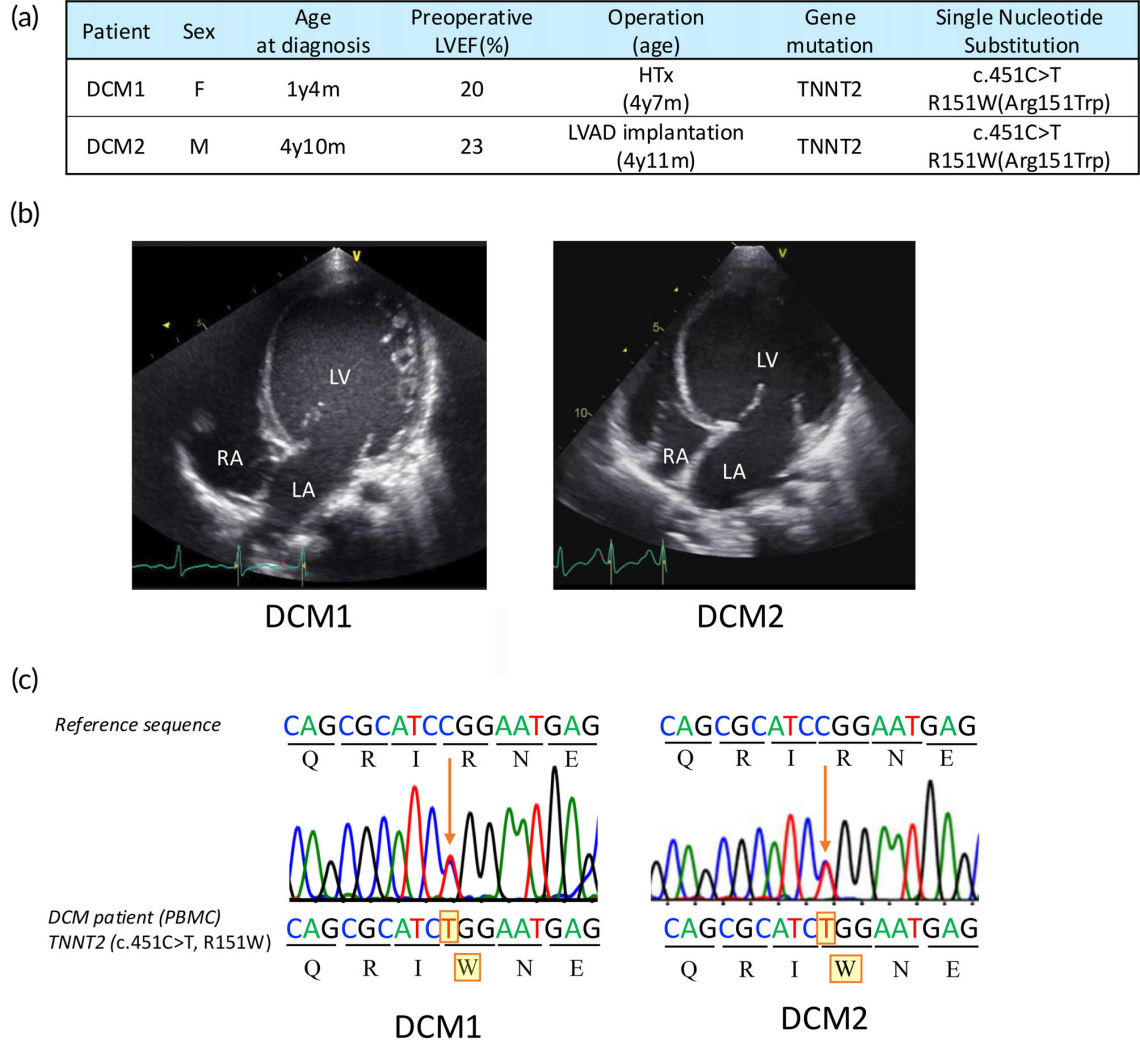
Characteristics of two pediatric DCM patients. (a) Clinical background of two pediatric DCM patients. (b) Transthoracic echocardiography of DCM1 and DCM2 revealed decreased left ventricular ejection fraction and a markedly dilated left ventricle. (c) Direct Sanger sequencing analysis of the TNNT2 locus using genomic DNA obtained from peripheral blood mononuclear cells.

**FIGURE 2:**
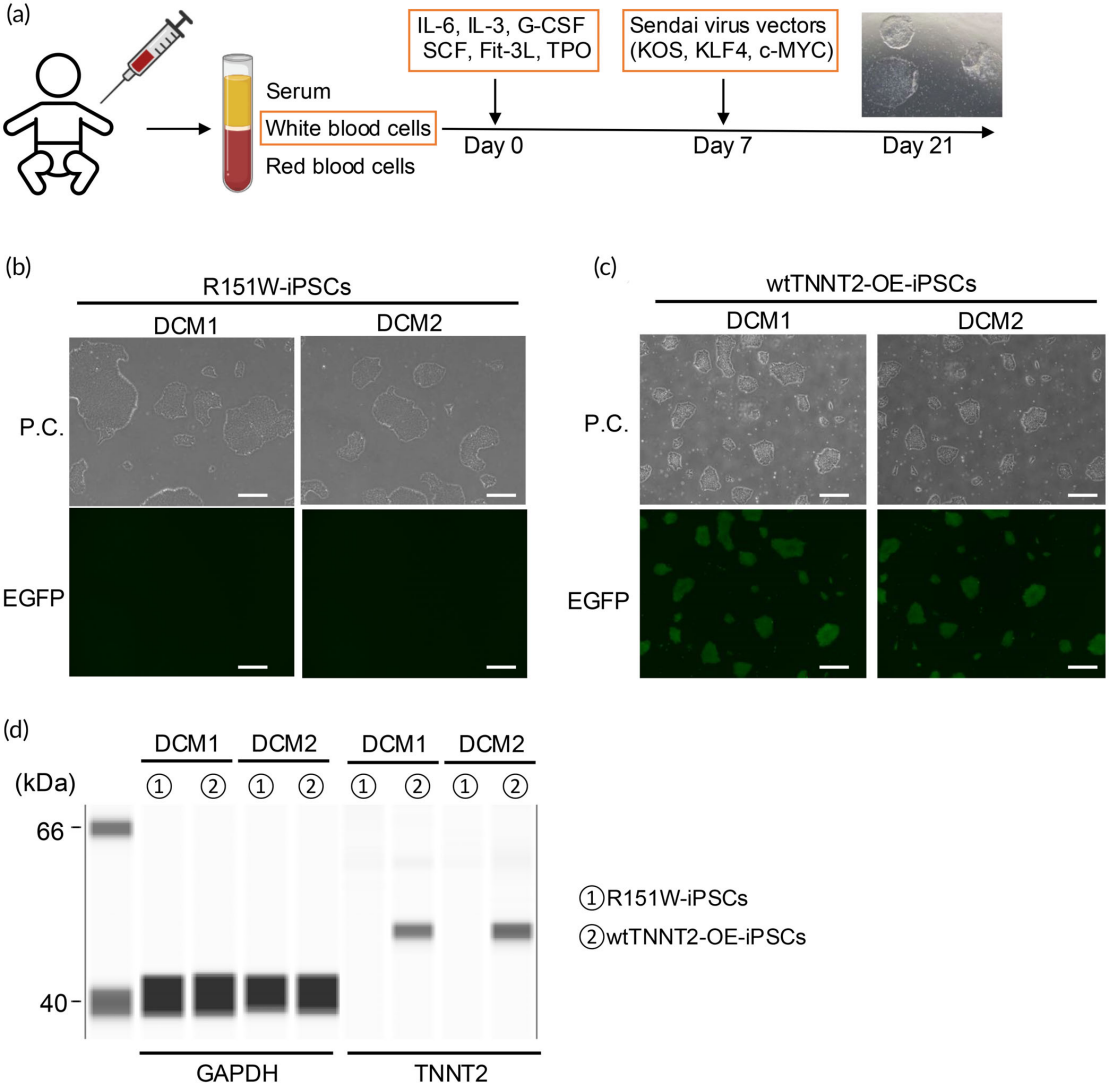
Generation of patient‐derived iPSCs. (a) Stepwise protocol for patient‐derived iPSC generation from PBMCs. (b) Representative images of R151W‐iPSCs. Scale bar: 500 μm. (c) Representative images of wtTNNT2‐OE‐iPSCs. Scale bar: 500 μm. (d) Representative simple western images of R151W‐iPSCs and wtTNNT2‐OE‐iPSCs using GAPDH and TNNT2 antibodies. IL‐6, interleukin‐6; IL‐3, interleukin‐3; G‐CSF, Granulocyte colony‐stimulating factor; SCF, stem cell factor; Flt‐3 L, FMS‐like tyrosine kinase 3 ligand; TPO, Thrombopoietin; KOS, KLF4 + OCT3/4 + SOX2; P.C., phase contrast.

We present two cases of pediatric DCM patients, the clinical backgrounds of which are summarized in Figure 1a. The first case is a 1‐year‐old girl (DCM1), who was diagnosed with DCM at 1 year of age and registered for heart transplantation at the same time. Transthoracic echocardiography revealed a markedly dilated left ventricle and a preoperative left ventricular ejection fraction (LVEF) of 20% (Figure 1b). Due to severe heart failure, she underwent heart transplantation at 4 years 7 months old. The second case is a 4‐year‐old boy (DCM2), who was diagnosed with DCM at a previous hospital and was referred to our hospital with symptoms of congestive heart failure. Transthoracic echocardiography revealed a markedly dilated left ventricle and impaired systolic function, with LVEF of 23% (Figure 1b). Due to progressive hemodynamic deterioration, he underwent a left ventricular assist device (LVAD) implantation on the day of admission. Neither patient had a notable family history of cardiomyopathy or related cardiac disorders. The whole exome sequencing identified a heterozygous missense mutation (c.451C>T; p.Arg151Trp; R151W) in the *TNNT2* gene in both patients, a finding that was subsequently confirmed by Sanger sequencing (Figure 1c). Patient‐derived iPSCs were generated from the PBMCs of both patients using Sendai virus vectors and will be referred to as R151W‐iPSCs hereafter (Figure 2a, b). Flow cytometric analysis showed that the generated iPSC lines were positive for the pluripotency markers OCT3/4, SOX2, and SSEA4 and exhibited a normal karyotype (Figure S1a, b).

To assess the pathogenicity of the R151W mutation in the same genetic background, iPSC lines overexpressing wild‐type TNNT2 were generated from the DCM1 and DCM2 iPSC lines, respectively. For the generation of the overexpression lines, a piggyBac transposon vector encoding TNNT2 and EGFP with an Internal Ribosome Entry Site (IRES) linker under the CAG promoter was used (Figure S2a). Three days after transfection, EGFP‐positive cells were sorted, and overexpressing iPSC lines were established by single‐cell cloning (Figure S2b). These iPSC lines were referred to as TNNT2 overexpression (wtTNNT2‐OE) clones. Robust green fluorescence was observed in wtTNNT2‐OE clones from both DCM1 and DCM2, confirming EGFP expression (Figure 2c). Flow cytometric analysis showed that both generated wtTNNT2‐OE‐iPSC lines were positive for the pluripotency markers OCT3/4, SOX2, and SSEA4 (Figure S1a). Western blotting was also performed to assess TNNT2 protein expression levels in both R151W‐iPSCs and wtTNNT2‐OE‐iPSCs in both DCM1 and DCM2, showing that TNNT2 protein expression was present only in the wtTNNT2‐OE clones (Figure 2d).

### 3.2 Cardiac differentiation

**FIGURE 3:**
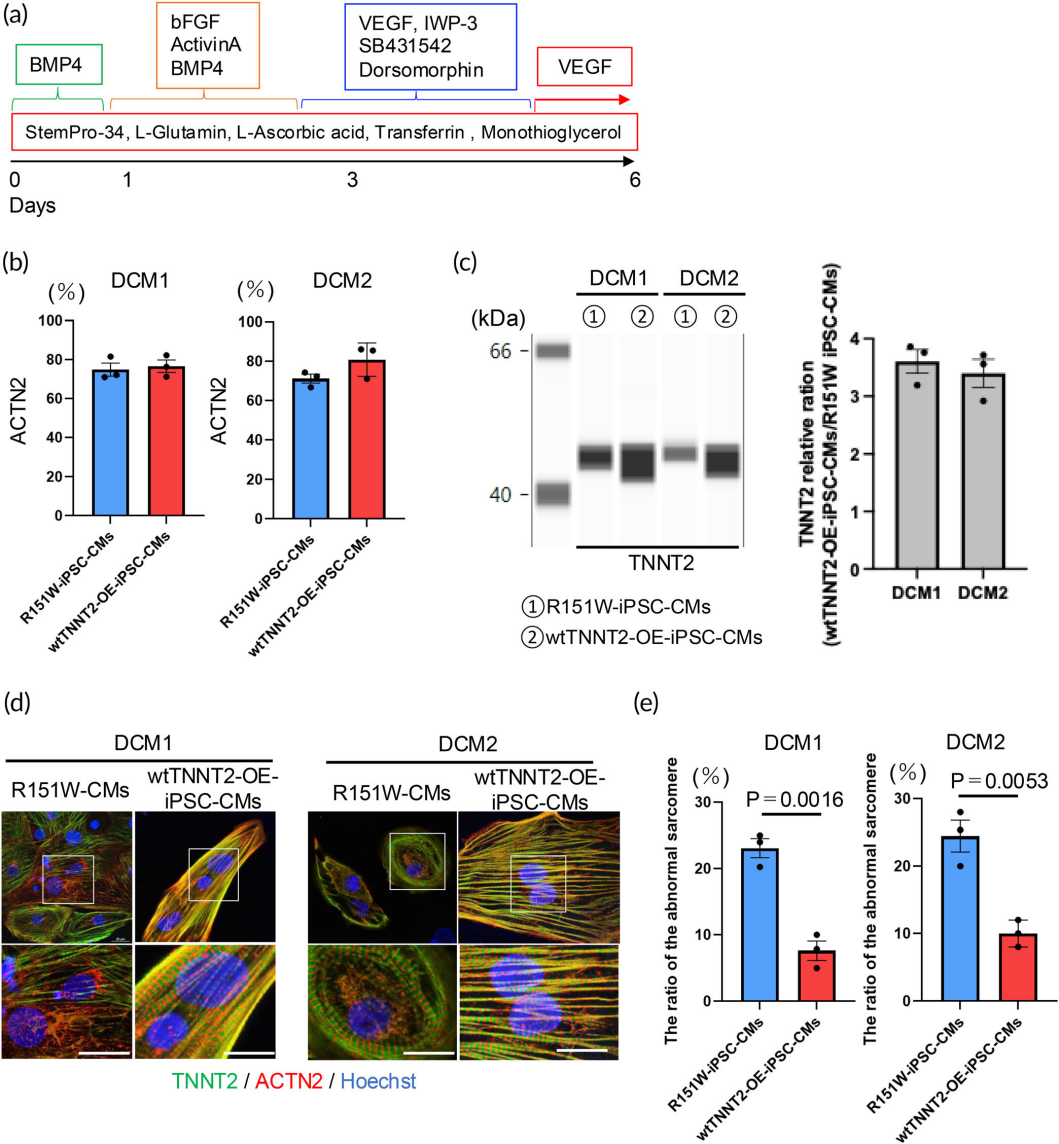
Cardiac differentiation. (a) Cardiac differentiation protocol. (b) ACTN2‐positive ratio in flow cytometric analysis of R151W‐iPSC‐CMs (*n* = 3) and wtTNNT2‐OE‐iPSC‐CMs (*n* = 3) for each DCM line. Data are presented as the mean ± SEM. (c) Left: Representative simple western images of TNNT2 protein expression in R151W‐iPSC‐CMs and wtTNNT2‐OE‐iPSC‐CMs derived from DCM1 and DCM2. Right: Relative TNNT2 expression in wtTNNT2‐OE‐iPSC‐CMs normalized to that in R151W‐iPSC‐CMs (set to 1.0) for each DCM line. Data are presented as mean ± SEM. (d) Representative immunofluorescence images of R151W‐iPSC‐CMs and wtTNNT2‐OE‐iPSC‐CMs stained with anti‐ACTN2 (red), anti‐Troponin T (green), and Hoechst (blue) for each DCM line. Scale bar: 20 μm. (e) The ratio of the abnormal sarcomeres in R151W‐iPSC‐CMs (*n* = 3) and wtTNNT2‐OE‐iPSC‐CMs (*n* = 3) for each DCM line. Data are presented as the mean ± SEM. Statistical analyses were performed using unpaired two‐tailed Student's *t* tests.

R151W‐iPSCs and wtTNNT2‐OE‐iPSCs were differentiated into CMs according to the protocol shown in Figure 3a with spontaneous beating observed around day 8 after induction of differentiation. EGFP expression persisted after cardiac differentiation (Figure S2c). Flow cytometric analysis was performed on day 14 to analyze differentiation efficiency using the ACTN2 antibody. The ACTN2‐positive rate was approximately 70%–80% in all iPSC lines, indicating efficient cardiac differentiation (Figure 3b). In addition, we quantified TNNT2 protein expression in R151W‐iPSC‐CMs and wtTNNT2‐OE‐iPSC‐CMs, finding that TNNT2 levels were consistently higher in wtTNNT2‐OE‐iPSC‐CMs than in R151W‐iPSC‐CMs for both DCM1 and DCM2 (Figure 3c). Next, we performed immunofluorescence staining on R151W‐iPSC‐CMs and wtTNNT2‐OE‐iPSC‐CMs. We observed sarcomere abnormalities in R151W‐iPSC‐CMs and a quantitative analysis confirmed that there was a significantly higher proportion of abnormal sarcomeric structures in R151W‐iPSC‐CMs derived from both DCM1 and DCM2 (Figure 3d, e). These results indicate that TNNT2 overexpression improved the number of abnormal sarcomeric structures.

### 3.3 R151W‐iPSC‐CMs were more Ca^2+^ desensitized than wtTNNT2‐OE‐iPSC‐CMs

**FIGURE 4:**
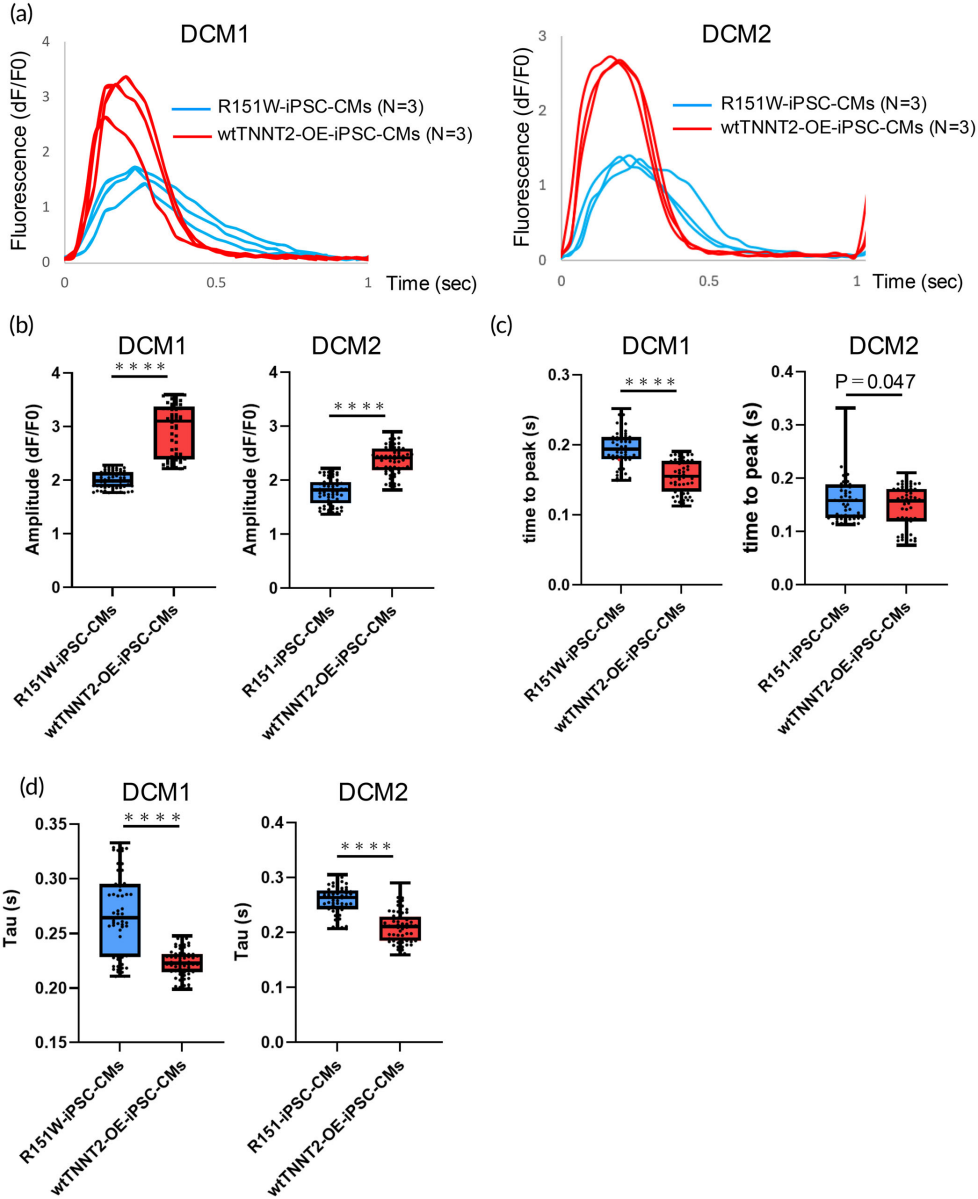
Ca^2+^ transients of R151W‐iPSC‐CMs and wtTNNT2‐OE‐iPSC‐CMs. (a) Representative recordings of the fluorescence signal of R151W‐iPSC‐CMs (*n* = 3) and wtTNNT2‐OE‐iPSC‐CMs (*n* = 3) from three independent experiments in DCM1 (left) and DCM2 (right) lines at a pacing rate of 1 Hz. (b–d) Amplitude (b), time to peak (c), and tau (d) were measured using high throughput fluorescence imaging at 1.0 Hz pacing (10 waves on average per region of interest [ROI]). *n* = 60 (R151W‐iPSC‐CMs) and *n* = 60 (wtTNNT2‐OE‐iPSC‐CMs) over three independent experiments. Boxes represent the 25th and 75th percentiles; whiskers represent the minimum and maximum ranges; horizontal lines indicate the median values. Statistical analyses were performed using unpaired two‐tailed Student's *t* tests. ****p < 0.0001.

We evaluated the intracellular Ca^2+^ kinetics in iPSC‐CMs using Calbryte™ 590 AM as a calcium indicator, while stimulating the CMs electrically at 1 Hz during recording. The parameters of amplitude, time to peak, and decay time constant (tau) are defined in Figure S2d. Figure 4a shows representative fluorescence signal waveforms in R151W‐iPSC‐CMs and wtTNNT2‐OE‐iPSC‐CMs. Evaluation of Ca^2+^ handling revealed a significant reduction in both amplitude and time to peak in R151W‐iPSC‐CMs, characteristic of CMs of the failing heart (Figure 4b,c). Furthermore, the decay tau, which is defined as the time required for an 80% reduction in signal, reflecting Ca^2+^ reuptake efficacy during relaxation, was also significantly prolonged in R151W‐iPSC‐CMs (Figure 4d). These properties were rescued in wtTNNT2‐OE‐iPSC‐CMs, suggesting that the R151W mutation causes abnormal calcium dynamics in vitro.

### 3.4 Contraction kinetics of R151W‐EHT and wtTNNT2‐OE‐EHT

**FIGURE 5:**
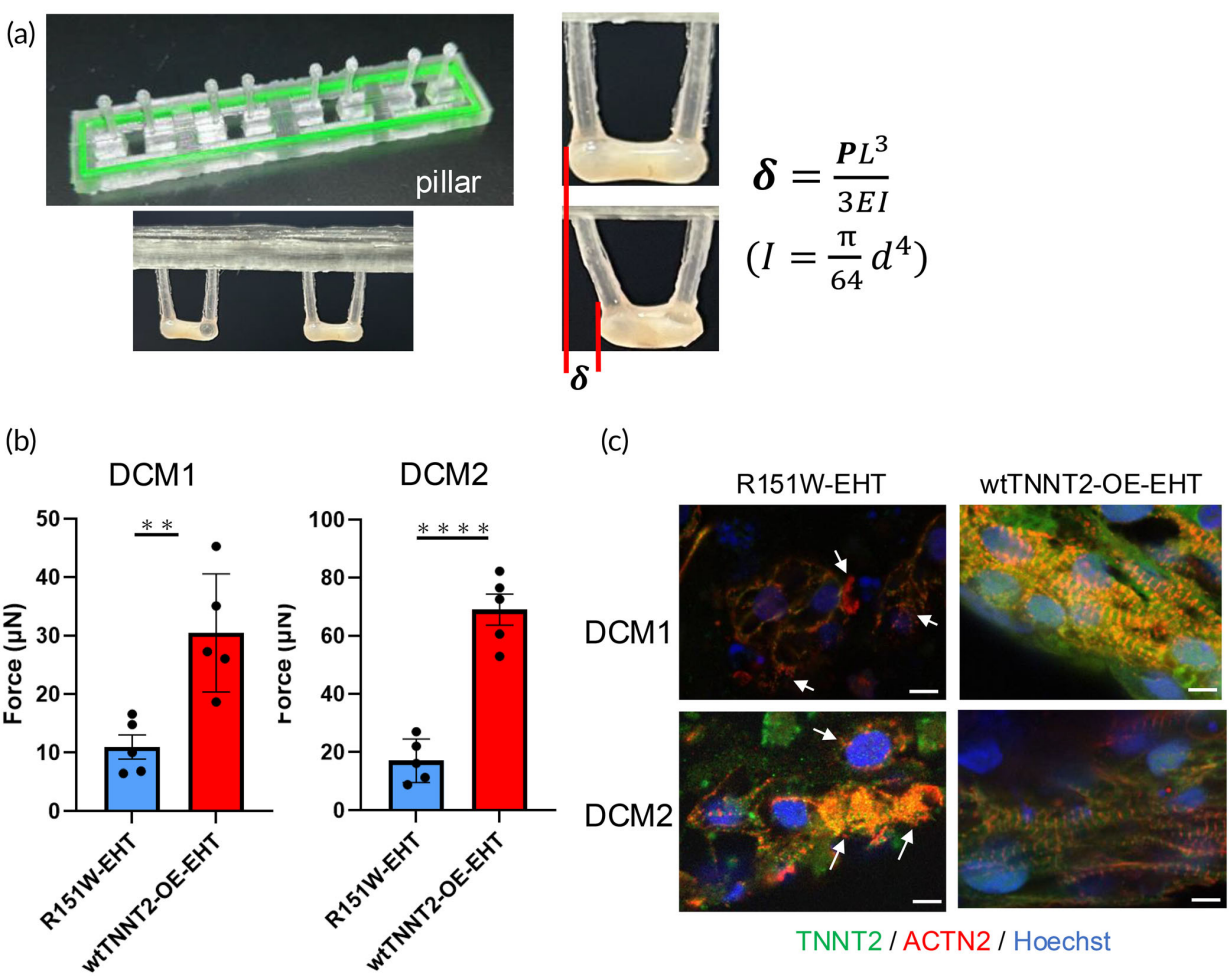
Fabrication of EHT using R151W‐iPSC‐CMs and wtTNNT2‐OE‐iPSC‐CMs. (a) Tissue pillars. The EHT pillar spacing was 6.5 mm. Side view of tissue attaching to the pillar after 2 weeks. (b) Measurement of the force at maximum pillar deflection of the EHT 2 weeks after tissue fabrication (*n* = 5 tissues per group). Data are presented as the mean ± SEM. Statistical analysis was performed using unpaired two‐tailed Student's *t* tests. ****p < 0.0001. **p < 0.001. (c) Representative immunofluorescence images of R151W‐iPSC‐EHT and wtTNNT2‐OE‐EHT stained with anti‐ACTN2 (red), anti‐Troponin T (green), and Hoechst (blue). Scale bar: 10 μm.

To investigate the contractile dynamics in the TNNT2 mutant DCM model, we fabricated EHTs using R151W‐iPSC‐CMs (R151W‐EHT) and wtTNNT2‐OE‐iPSC‐CMs (wtTNNT2‐OE‐EHT) (Figure 5a). The EHT initiated contractions after approximately 4–5 days of culture. Subsequently, on day 14, pillar deflection data were recorded. To assess the kinetics of EHT contraction, we used video microscopy to track one‐dimensional pillar deflection under 1 Hz stimulation. The distance of the pillar head movement resulting from EHT beating was gauged, and the force (N) was derived by calculating the distance of movement along with the material data of the pillar. The force formula relies solely on the change in the pillar's displacement as the only parameter. The force at maximum pillar deflection is shown in Figure 5b. R151W‐EHTs showed weaker contraction than wtTNNT2‐OE‐EHTs in both DCM1 and DCM2 (Videos S1, S2, S3, S4). In addition, immunofluorescence analysis of the EHTs demonstrated abnormal sarcomeric structures in R151W‐EHT cells, resembling that observed in R151W‐iPSC‐CMs (Figure 5c). These data suggest that our EHT model associated with the TNNT2‐R151W mutation reflects the clinical manifestations of DCM with systolic dysfunction.

## 4 DISCUSSION

In this study, two iPSC lines were generated from two unrelated pediatric DCM patients harboring the same TNNT2‐R151W mutation. The resulting patient‐specific iPSC‐CMs were characterized in vitro and compared with iPSC‐CMs that were engineered to overexpress wild‐type TNNT2 protein using their iPSC lines. Ca^2+^ handling analyses revealed that R151W‐iPSC‐CMs exhibited both a significant reduction in peak fluorescence intensity and a prolonged time to peak fluorescence, which are characteristic of failing heart tissue. They also exhibited a markedly delayed Ca^2+^ reuptake during relaxation. Additionally, R151W‐iPSC‐CMs displayed abnormalities in sarcomere alignment. Furthermore, we fabricated EHT using iPSC‐CMs and evaluated their contractile dynamics in vitro. R151W‐EHT exhibited significantly reduced contractile force compared to wtTNNT2‐OE‐EHT.

The R151W mutation in the TNNT2 gene is the same single‐nucleotide mutation (Chr1:201364336 G>A, GRCh38) that was previously reported as R141W.^33^ This discrepancy arises from the use of an alternative TNNT2 transcript reference (NM_001276345.2). To our knowledge, this is the first study to model the TNNT2 R151W variant in pediatric DCM using patient‐derived hiPSCs and EHTs. The TNNT2‐R141W mutation in the mouse model has been reported in several studies as a cause of DCM and is associated with Ca^2+^ desensitization and reduced contractility.^34^, ^35^ Transgenic mice expressing the TNNT2‐R141W variant exhibit left ventricular dilatation and impaired contractility.^34^ Ramratnam M. et al. demonstrated that hearts harboring the TNNT2‐R141W variant exhibit increased peak systolic and end‐diastolic intracellular Ca^2+^ concentrations, as well as prolonged Ca^2+^ uptake and decay kinetics.^35^ In vitro motility assays revealed that thin filament mutations containing the TNNT2 R141W mutation, associated with DCM, abolish the relationship between myofilament Ca^2+^ sensitivity and Troponin I phosphorylation by protein kinase A.^36^ Our Ca^2+^ handling analysis yielded analogous results, implicating the R151W mutation in a similar uncoupling of myofilament Ca^2+^ sensitivity from Troponin I phosphorylation by protein kinase A. Consistently, Xu et al. introduced the R141W mutation into wild‐type healthy iPSCs using CRISPR/Cas9 and reported that resultant iPSC‐CMs displayed markedly reduced contractile force and prolonged contractile‐relaxation cycles at the single cell level.^37^ These results are consistent with our observations.

We fabricated our EHT model to evaluate contraction force using our previously described method.^32^ In this system, the deflection of the pillar is the only variable and is directly proportional to the force generated by the EHT. This enables us to measure force waveforms from the video of pillar movement. Our results showed that R151W‐EHT produced significantly lower contractile forces than wtTNNT2‐OE‐EHT. The observed difference in contractile force between wtTNNT2‐OE‐EHT derived from DCM1 and DCM2 may be due to a combination of factors, including differences in TNNT2 expression levels, EHT maturity, and genetic and epigenetic modifiers. In our EHT model, we detected TNNI1 expression, indicating the presence of the fetal cardiac troponin I isoform, as well as TNNI3 expression, indicating the presence of the adult troponin I isoform (Figure S3a). These results imply that our in vitro model could evaluate cardiac tissue transitioning from the prenatal to neonatal stage, in which TNNI1 and TNNI3 coexpress.^38^ Furthermore, wtTNNT2‐OE‐iPSC‐CMs exhibit approximately a threefold increase in TNNT2 expression (Figure 3c). These findings suggest that the increased wild‐type/mutant TNNT2 ratio in these iPSC‐EHTs, which have the same genetic background, could be the reason for the improvement in contractility and sarcomere structure. These observations also suggest that our EHT model might mimic the systolic dysfunction observed in patients with TNNT2 mutation‐associated pediatric DCM.

Hasegawa et al. have demonstrated that our EHT platform can also evaluate diastolic dysfunction in restrictive cardiomyopathy, highlighting its broader applications.^32^ However, several limitations should be acknowledged. First, the forces are measured only along the longitudinal direction of the pillar, which may not capture the multidirectional mechanics of the human ventricle. Second, this model does not reflect left ventricular dilatation, which is a significant characteristic of DCM. Recently, Chang H. et al. manufactured helically and circumferentially aligned tissue‐engineered models of the left ventricle using focused rotary jet spinning.^39^ This technology can assess left ventricular enlargement, which was previously difficult to replicate and measure forces in myocardial cells beyond the longitudinal direction. In the future, this technology could be applied to DCM models, resulting in more accurate simulations.

Gene therapy is a promising strategy for treating a wide range of human diseases because it addresses the underlying molecular cause.^40^ Therapeutic approaches include gene replacement, which provides exogenous functional cDNA and genome editing, which corrects pathogenic variants or modulates regulatory elements in situ.^41^ In recent years, adeno‐associated viral (AAV)‐mediated gene replacement strategies have advanced rapidly toward clinical development, offering a promising new approach to treating cardiac diseases. Clinical translation is already underway. A first‐in‐human phase 1 trial has demonstrated the safety and preliminary efficacy of systemic AAV9‐LAMP2B delivery in patients with Danon disease‐associated hypertrophic cardiomyopathy.^42^ However, gene therapy for DCM is still in the preclinical stage. Loss of function variants in BAG3 are well‐recognized drivers of DCM,^43^ and intravenous AAV9 BAG3 delivery forestalled ventricular dilatation and dysfunction in Bag3^+/−^ mice.^44^ Additionally, low doses of AAV9‐BAG3 were administered in miniature pigs via a catheter‐based retrograde coronary sinus infusion, resulting in extensive transduction of the myocardium.^45^

In this study, we demonstrated that overexpression of wild‐type TNNT2 in patient‐derived iPSC‐CMs carrying the R151W mutation restores sarcomere structure, normalizes Ca^2+^ handling, and rescues contractile force. These findings support the feasibility of AAV‐based gene replacement therapy as a strategy to restore cardiac function in DCM patients with the TNNT2‐R151W mutation and related pathogenic variants.

## 5 CONCLUSIONS

Our study demonstrated that the R151W‐iPSC‐CMs exhibited Ca^2+^ desensitization, abnormal sarcomere structure, and impaired contractility. These properties were ameliorated in wtTNNT2‐OE‐iPSC‐CMs, indicating that R151W‐iPSC‐CMs reflect DCM phenotypes in vitro. These results suggest that gene therapy with normal TNNT2 may represent a promising therapeutic approach for pediatric DCM patients with TNNT2 mutations.

## AUTHOR CONTRIBUTIONS

Toshiaki Nagashima, Kenji Miki, Hidekazu Ishida, Kunio Kashino, and Shigeru Miyagawa conceived and designed the project. Toshiaki Nagashima, Kenji Miki, Masaru Tsuchida, Ikue Takei‐Sasozaki, and Yuki Higashiyama performed the experimental work. Toshiaki Nagashima, Kenji Miki, Julia Whitehouse, and Shigeru Miyagawa interpreted the data and wrote the manuscript. All the authors discussed the results.

## CONFLICT OF INTEREST STATEMENT

The authors declare no conflicts of interest.

## Supporting information


**Figure S1:** Characteristics of R151W‐iPSCs.
**Figure S2:** Generation and analysis of wtTNNT2‐OE‐iPSCs.
**Figure S3:** Analysis of TNNI isoform in EHTs.

**Video S1:** Supplementary video 1.

**Video S2:** Supplementary video 2.

**Video S3:** Supplementary video 3.

**Video S4:** Supplementary video 4.

## Data Availability

The data that supports the findings of this study are available in the supplementary material of this article.
