# Supplementary material for: Pediatric dilated cardiomyopathy caused by TNNT2‐R151W mutation: Modeling and rescue in patient‐derived induced pluripotent stem cells and engineered heart tissue
Source: Bioeng Transl Med. 2026 Jan 6;11(2):e70108. doi: 10.1002/btm2.70108 (PMC13093569; doi:10.1002/btm2.70108)
Supplement: Supplementary file 1 — Figure S1: Characteristics of R151W‐iPSCs. Figure S2: Generation and analysis of wtTNNT2‐OE‐iPSCs. Figure S3: Analysis of TNNI isoform in EHTs. [file BTM2-11-e70108-s004.docx]

**Figure S1 Characteristics of R151W-iPSCs.**

a. Flow cytometry analysis of the pluripotency markers OCT3/4, SOX2, and SSEA4 in R151W-iPSCs (left) and wtTNNT2-OE-iPSCs (right). The red trace in each histogram represents stained samples for the respective marker and demonstrates that most cells are positive for the marker. The gray graph indicates unstained controls.

b. The karyotypes of R151W-iPSCs of DCM1 and DCM2 showed normal pattern.

**Figure S2 Generation and analysis of wtTNNT2-OE-iPSCs.**

a. Construction of the piggyBac transposon vector.

b. Generation of wtTNNT2-OE-iPSCs cells from R151W-iPSCs by transfection, sorting and single-cell cloning.

c. Representative images of wtTNNT2-OE-iPSC-CMs of DCM1 and DCM2. Scale bar: 500 μm.

d. Amplitude, time to peak and tau are defined in the graph of Ca^2+^ transients.

*P.C., Phase Contrast*

**Figure S3** Analysis of TNNI isoform in EHTs.

a. Representative simple western images of TNNI3 and TNNI1 protein expression in R151W-EHT and wtTNNT2-OE-EHT derived from DCM1 and DCM2.
